# Sixth Decennial Pharmacopœia Convention

**Published:** 1880-02

**Authors:** James E. Morgan

**Affiliations:** 905 E Street, N. W., Washington, D. C.


### SIXTH DECENNIAL PHARMACOPŒIA CONVENTION.

By virtue of authority devolved upon me as the last surviving officer of the Pharmacopœia Convention of 1870, I again call the attention of "the several incorporated State medical societies, the incorporated medical colleges, the incorporated colleges of physicians and surgeons, and the incorporated colleges of pharmacy througuout the United States," to the importance of sending delegates to the Sixth Decennial Pharmacopœia Convention, and of sending the names and residences of the same to me for publication. The Convention meets on the first Wednesday in May, 1880, and I am required "to publish the names and residences of the delegates, for the information of the medical public, previous to its meeting."

I have received so far the names and residences of the following delegates:

From the Massachusetts College of Pharmacy, Boston: Prof. G. F. H. Markoe, Ph.G.; Samuel A. D. Sheppard, Ph. G.; Thomas Doliber, Ph.G.

From the Philadelphia College of Pharmacy: Prof. John M. Maisch, Alfred B. Taylor, Prof. Joseph P. Remington.

From the Louisville College of Pharmacy: Prof. Emil Scheffer, Prof. C. Lewis Deihl, E. Vincent Davis.

From the Maryland College of Pharmacy, Baltimore: Delegates—Wm. S. Thomas, Louis Dohme, Joseph Roberts. Alternates—Charles Caspari, Jr., Dr. John F. Moore, Dr. Robert Lautenbach.

From the Medical Society of the District of Columbia: Prof. D. W. Prentiss, M.D.; Prof. Thomas Antisell, M.D.; Emeritus Prof. James E. Morgan, M.D.

From the National Medical College of Columbia University, Washington, D. C.: Prof. W. W. Johnston, M.D.; Prof. D. W. Prentiss, M.D.

From the Medical Department of the University of Georgetown, D. C.: Prof. W. H. Ross, M.D.; Prof. C. H. A. Kleinschmidt, M.D., and Dr. S. C. Busey.

From the National College of Pharmacy, Washington,

D. C.: Mr. W. S. Thomson, Prof. Oscar Oldberg, Mr. R. B. Ferguson.

[Signed] JAMES E. MORGAN, M.D.,
905 E Street, N. W., Washington, D. C.

WASHINGTON, D. C., Jan. 28, 1880.

